# Interleukin-6: A Constitutive Modulator of Glycoprotein 130, Neuroinflammatory and Cell Survival Signaling in Retina

**DOI:** 10.4172/2155-9899.1000439

**Published:** 2016-07-22

**Authors:** Franklin D. Echevarria, Abigayle E. Rickman, Rebecca M. Sappington

**Affiliations:** 1 Neuroscience Graduate Program, Vanderbilt University, Nashville, TN, USA; 2 Vanderbilt Eye Institute, Vanderbilt University Medical Center, Nashville, TN, USA; 3 Department of Ophthalmology and Visual Sciences, Vanderbilt University School of Medicine, Nashville, TN, USA; 4 Department of Pharmacology, Vanderbilt University School of Medicine, Nashville, TN, USA

**Keywords:** Gp130, Interleukin-6, Inflammation, Retina, Glaucoma

## Abstract

**Objective:**

The interleukin-6 (IL-6) family of cytokines and their signal transducer glycoprotein (gp130) are implicated in inflammatory and cell survival functions in glaucoma. There are several avenues for interdependent modulation of IL-6 family members and gp130 signaling. Here we investigated whether IL-6 modulates gp130 and related neuroinflammatory, cell survival and regulatory signaling in both healthy and glaucomatous retina.

**Methods:**

In naïve and glaucomatous (Microbead Occlusion Model), wildtype (WT) and IL-6 knockout (*IL-6−/−*) mice, we examined gp130 protein expression and localization, using western blot and immunohistochemistry. Gene targets related to IL-6 and gp130 signaling and pertinent to neuroinflammation (TNFα, IL-1β), cell health (Bax, Bcl-xl) and STAT3 regulation (Socs3) were quantified using qRTPCR.

**Results:**

In the naïve retina, *IL-6−/−* retina contained significantly less gp130 compared to WT retina. This IL-6-related decrease in gp130 was accompanied by a reduction in mRNA expression of *TNFα*, *Socs3* and *Bax*. After 4 weeks of microbead-induced ocular hypertension, both microbead- and saline-injected (control) eyes of *IL-6−/−* mice exhibited higher expression of *TNFα*, compared to WT mice. *IL-1β* expression was also reduced specifically in *IL-6−/−* retina with microbead-induced glaucoma. While saline and microbead injection increased *Bcl-xl* and *Socs3* mRNA in both WT and *IL-6−/−* mice, *IL-6−/−* deficiency led to smaller increases for both *Bcl-xl* and *Socs3*.

**Conclusions:**

Our findings support a role for IL-6 in setting baseline parameters for neuroinflammatory, cell health and gp130 regulatory signaling that can impact the nature and magnitude of retinal responses to glaucoma-related stressors.

## Introduction

**Figure 1:**
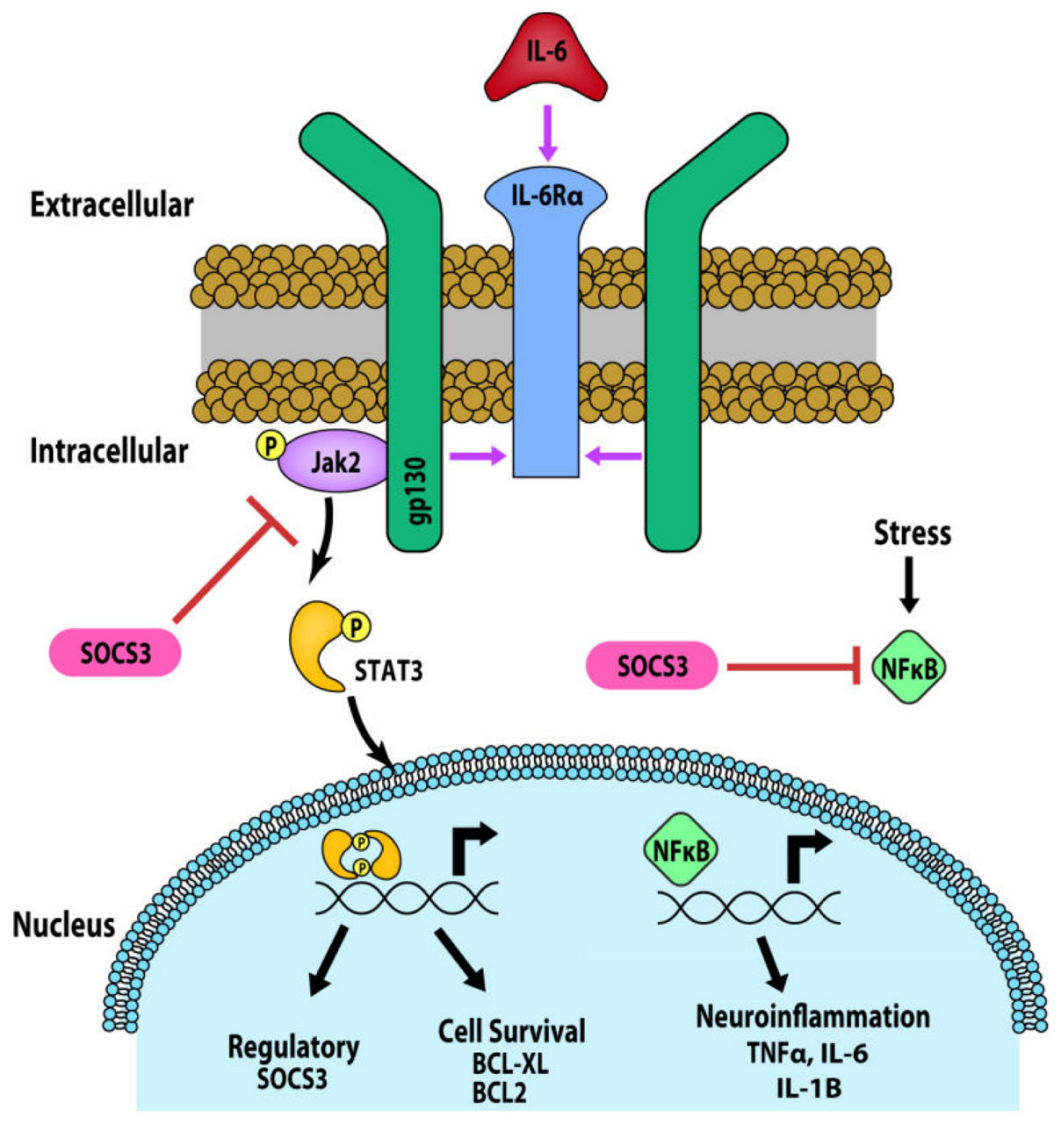
Review of IL-6 signaling. Under naïve and pathological conditions, IL-6 binds to IL-6Rα, followed by recruitment of signal transducer gp130 (purple arrows). Upon formation of the IL-6/IL-6Rα/gp130 complex, gp130 phosphorylates Jak2, followed by subsequent activation/phosphorylation of STAT3. Phosphorylated STAT3 dimerizes and translocates to the nucleus to drive transcription of genes associated with cell survival (Bxl-XL, BCL-2) and gp130 inhibitory feedback (Socs3). Socs3 can also act to inhibit NFκB, which regulates the expression of inflammatory cytokines (i.e. IL-6, IL-1β, TNFα).

IL-6 mediates diverse cellular functions that are not limited to pathological inflammation [1]. IL-6 is part of a larger group of cytokines called the “IL-6” or “gp130” family of cytokines that includes ciliary neurotrophic factor (CNTF), interleukin-11 (IL-11), oncostatin M (OSM), leukemia inhibitory factor (LIF), cardiotrophin-like cytokine factor-1 (CLCF1), cardiotrophin-1 (CTF-1) and cardiotropin-2 (CTF-2). Members of the IL-6 cytokine family are defined by a common signal transduction pathway, which is initiated by gp130. To signal, each family member binds a respective alpha receptor (e.g. IL-6 binds to IL-6Rα), which then recruits and activates gp130 (Figure 1). Activation of gp130 leads to a myriad of signal transduction cascades, including JAK/STAT, that drives transcription of genes associated with cell health and survival, neuroinflammation and regulation of gp130-mediated signaling (Figure 1) [1,2].

The shared mechanism of signal transduction produces functional redundancy among IL-6 family members [1]. For example, IL-6 protects retinal ganglion cells (RGCs) from apoptosis and promotes axon regeneration following optic nerve crush [3,4]. Other IL-6 family members, including CNTF, IL-11, OSM and CLCF1, are also associated with optic nerve regeneration [5–7]. Recent literature indicates that gp130-mediated activation of JAK/STAT pathways, which can be accomplished with all IL-6 family members, is a modulator of axon regeneration in the optic nerve [7,8]. Across several studies, we determined that the IL-6 family of cytokines is also relevant for RGC degeneration in glaucoma. Glaucoma is a chronic form of RGC degeneration that is associated with both age and sensitivity to intraocular pressure (IOP). In our studies, we determined that elevated IOP alters the expression of nearly all members of the IL-6 family of cytokines as well as gp130 [9,10]. We also demonstrated that pressure-induced production of IL-6 is dependent upon NFκB activation and promotes survival of RGCs through induction of immediate early genes [11,12]. Other groups have reported similar findings, including pro-survival functions for other IL-6 family members, i.e. CNTF, in glaucoma [13,14].

Functional redundancy relates not only to outcomes of neuroinflammation and cell survival, but also to feedback pathways that positively and negatively regulate gp130 signaling. For example, SOCS3 is a downstream target of gp130 signaling that inhibits JAK/STAT activity to establish a negative feedback loop [15,16]. This type of regulatory signaling provides an avenue for IL-6-related cytokines to impact future gp130-mediated responses to any or all members of the cytokine family. When considering the role of the IL-6 family in neuroinflammation and cell survival, regulatory outcomes have the potential to broadly impact the initiation and propagation of stress responses. Of particular interest is that IL-6 and several of the other IL-6 family members are constitutively expressed at high levels in retina [9,10]. In this context, regulatory signaling that mediates gp130 activity may be relevant for establishing baseline parameters for inflammation and cell health and the subsequent capacity for stress response.

Using *IL-6−/−* mice and our inducible, microbead occlusion model of ocular hypertension, we aim to determine whether IL-6 signaling plays a role in: 1) establishing baseline parameters for gp130-mediated signaling, neuroinflammation and cell health and 2) defining the nature and magnitude of IOP-induced neuroinflammatory, cell health and gp130-related regulatory signaling.

## Methods

### Animals

Adult *IL-6−/−* mice (B6.129S2-IL6tm1kopf/J) and respective wildtype (WT; B6129SF2/J) controls were used for all experiments. Breeder pairs were originally obtained from Jackson Laboratories (Bar Harbor, ME) and experimental mice were bred and genotyped in-house, using primers provided by Jackson Labs. Mice were housed in accordance with NIH guidelines and maintained on a 12 hr light/dark cycle with free access to food and water. All experiments complied with the ARVO statement for the use of animals in ophthalmic and vision research and were approved by the IACUC of Vanderbilt University Medical Center.

### Microbead occlusion of trabecular meshwork and IOP measurements

IOP elevation for 4 weeks was induced in adult male WT and *IL-6−/−* mice using the microbead occlusion model, as previously described [17]. Briefly, anesthetized mice received a unilateral injection of 1.5 ul (1 × 10^6^ microbeads/mL) of 15 μm polystyrene beads (Cat# F8844; Life Tech) in one eye. The contralateral eye served as a surgical control and was injected with an equal volume of saline. IOP was measured in awake, behaving mice, using a Tonolab tonometer (TonoLab; Reichert, Depew, NY) [16,18,19]. Prior to initial injections, mean baseline IOP was calculated from approximately 60 readings taken over a period of 10–14 days. Following microbead and saline injections, IOP was determined as the mean of 20–30 measurements, taken every 2–3 days throughout the 4 week study.

### Tissue preparation

For histological experiments, mice were sacrificed by transcardial perfusion as previously described [9,20]. Eyes were stored in 4% PFA at 4°C until use. Retinas were cryopreserved in 30% sucrose and 10 μm cryostat sections were obtained following embedding in OCT medium. For western blot and PCR experiments, mice were sacrificed by cervical dislocation followed by decapitation. Eyes were enucleated, retinas dissected, snap frozen on dry ice and stored at −80°C until use.

### Immunohistochemistry and imaging

Labeling of whole-mount retina and retinal cryo-sections were done as previously described [11,16]. Primary antibodies used were gp130 (1:200; Cat# MAB4681, R&D Systems) and β-Tubulin III (TUJ1, 1:500; Cat#845501; BioLegend). Secondary antibodies were used at a concentration of 1:200 and consisted of donkey α-rat 488 (cat# 712-545-150; Jackson Immuno) or donkey α-rabbit 647 (cat# 711-606-152; Jackson Immuno). Z-stack images (0.5 μm/stack) were obtained on an inverted confocal microscope (Olympus) at the Vanderbilt University Cell Imaging Services Core and were analyzed with FV-10 ASW image analysis software (Olympus).

### Immunoblotting

Retina was homogenized in lysis buffer containing: 50 mM Tris HCl, 150 mM NaCl, 1% NP-40, 0.25% Sodiμm Deoxycholate, 100 μm PMSF, protease inhibitor cocktail tablet (Cat# 04693159001; Roche) and Phosphatase Inhibitor Cocktail Tablet (Cat# 04906845001; Roche). Protein concentration was determined with BCA Assay (Cat# 23225; Thermo Fisher Scientific). 20 μg of sample protein was mixed with Laemmli Sample Buffer (Cat# 161-0747; BioRad) containing 10% βME (Cat# M3148; Sigma), separated by SDS-Page in a 4–20% gradient Tris-glycine pre-cast gel (Cat# 345-0032; BioRad) and transferred to a nitrocellulose membrane (Cat# 45004004; GE Healthcare). Membrane was incubated in Odyssey Blocking buffer (Cat# 927-50000; Licor) overnight at 4°C followed by overnight incubation at 4°C in blocking buffer containing 0.2% Tween-20, anti-rabbit gp130 (1:50; cat# sc-655; Santa Cruz) and anti-mouse β-actin (1:1000; cat# AM4302; Ambion). Following washes in 1XTBS with 0.2% Tween-20, membranes were incubated at RT for 1 hour in blocking solution containing: 0.2% Tween-20, 10% SDS, donkey anti-mouse IRDye 680RD (1:1000; Cat# 925-68072; Li-Cor) and donkey anti-rabbit IRDye 800CW (1:1000; Cat# 925-32213; Li-Cor). Immuno-reactive bands were detected using the Odyssey infared imaging system (Li-Cor; Lincoln, NE). Gp130 bands (130 kD) were quantified by densitometry analysis (Odyssey Application Software V3.0; Li-Cor) and normalized to β-actin bands (42 kD). To ensure accurate comparisons between WT and *IL-6−/−*, samples from both genotypes were run on the same gel and transferred to the same membrane.

### RNA isolation and DNase treatment

RNA was isolated using TRIzol (Cat# 15596026; Life Tech) followed by chloroform purification, as per manufacturer’s recommendations. RNA concentration was measured using a nanodrop 2000 spectrophotometer (Ambion). To remove any contaminating DNA, 1 μg of RNA was subjected to DNase treatment according to manufacturer’s instructions (Cat# 18068-015; Invitrogen). Purified RNA was stored at −80°C or directly used for cDNA synthesis.

### cDNA synthesis and qRTPCR

cDNA synthesis and qRTPCR was performed as previously described [9]. Briefly, 1 μg of RNA was converted to cDNA using the Superscript VILO cDNA Synthesis kit (Cat# 11754-050; Invitrogen) according to manufacturer’s instructions. cDNA samples were placed at −20°C or used directly for qRTPCR. To measure gene expression, 50 ng cDNA was added to a solution containing: Taqman Gene Expression master mix (Cat# 4369016; ThermoFischer), Taqman Gene Expression Probe and ddH2O. Each sample was run in triplicate. Taqman probes used were *gp130* (Mm00439665_m1), *IL-1β* (Mm00434228_m1), *TNFα* (Mm00446190_m1), *BCL-XL* (Mm00437783_m1), *BAX* (Mm00432051_m1), *SOCS3* (Mm00545913_s1) and *Gapdh* (Mm99999915_g1). qRTPCR was run in a 7300 Real Time PCR System. To further confirm suitability of samples, a 1 μl of RNA from each sample was reserved to serve as a – RT negative control for gene-specific qPCR. The threshold of cycle (Ct) values for each gene was analyzed using SDS software (Applied Biosystems) and subtracted by the Ct value for GAPDH in each. To determine gene expression differences between WT and *IL-6−/−* in naïve samples, *IL-6−/−* samples were normalized to WT using the ΔΔCt method, as indicated by probe efficiencies. For microbead samples, saline and microbead retinas from WT and *IL-6−/−* were normalized to respective genotype naïve samples.

### Statistical analysis

Data is represented either as mean ± STDEV or mean ± SEM. Statistical analysis was performed using SigmaPlot (Systat Software Inc., San Jose, CA). Depending on the comparison, statistical significance was determined using student’s t-test, Wilcoxon Signed Rank test, paired t-test, One-sample t-test or a one-way ANOVA with Bonferroni pairwise comparisons. Normality (Shapiro-Wilk) and equal variance was also assessed for each comparison. For all experiments, p<0.05 was considered statistically significant.

## Results

### IL-6 mediates constitutive expression of gp130

**Figure 2:**
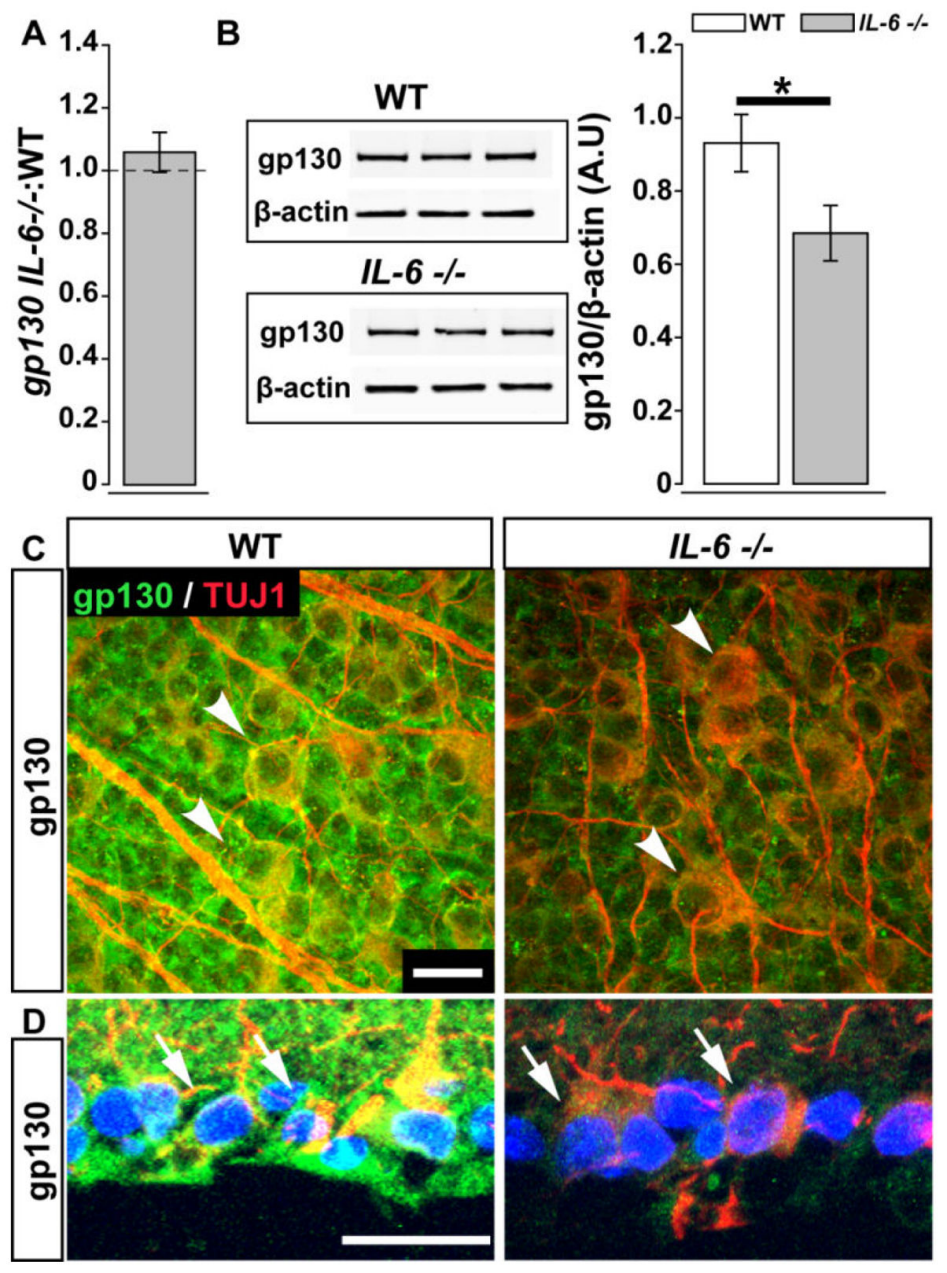
IL-6 deficiency leads to decreased gp130 protein in the retina. (**A**) Fold difference of *Gp130* mRNA in the *IL-6−/−* retina normalized to WT *Gp130* mRNA (dotted line) via the ΔΔCt method. (**B**) Densitometry analysis (right) of gp130 and β-actin immunoblotting (left) in WT (white) and *IL-6−/−* (gray) retina.; student’s t-test. Error bars indicates STDEV. (**C–D**) Whole mount (**C**) and sagittal cross sections (**D**) of WT (left) and *IL-6−/−* (right) retina reveal co-localization (arrowheads; C and arrows; D) of gp130 immunolabeling (green) with β-Tubulin+ RGCs. Error bars represent standard deviation and asterisks indicate p<0.05 (**A,B**). Scale bars = 20 μm (**C,D**).

IL-6 and its signal transducer gp130 are constitutively expressed in murine retina [9,10]. Previous studies indicate that IL-6 can modulate gp130 expression in a negative feedback loop [15]. To determine whether IL-6 constitutively modulates gp130 expression in retina, we examined gene and protein expression of gp130 in retina from IL-6- deficient mice (*IL-6−/−*). Using quantitative RT-PCR, we did not detect a change in the expression level of gp130 mRNA in whole retina from *IL-6−/−* mice, as compared to WT mice (p>0.05; Figure 2A). However, western blot analysis revealed that gp130 protein decreases by 36% in whole retina lysates from *IL-6−/−* mice compared to WT mice (p<0.05; Figure 2B). Decreased protein expression of gp130 in *IL-6−/−* mice was also apparent in sagittal sections and wholemounted retina co-immunolabeled with antibodies against gp130 and the RGC marker β–tubulin (Figures 2C–2D). Consistent with our previous findings [9], gp130 immunolabeling was robust in the ganglion cell layer of WT retina and co-localized with β–tubulin-positive RGCs (Figures 2C–2D). In *IL-6−/−* retina, the localization pattern of gp130 immunolabeling was unaltered, but the intensity of labeling was greatly diminished (Figures 2C–2D). Together, these data suggest that IL-6 constitutively mediates expression of gp130 in murine retina.

### IL-6 deficiency alters baseline parameters for inflammatory, cell health and gp130 regulatory pathways

**Figure 3:**
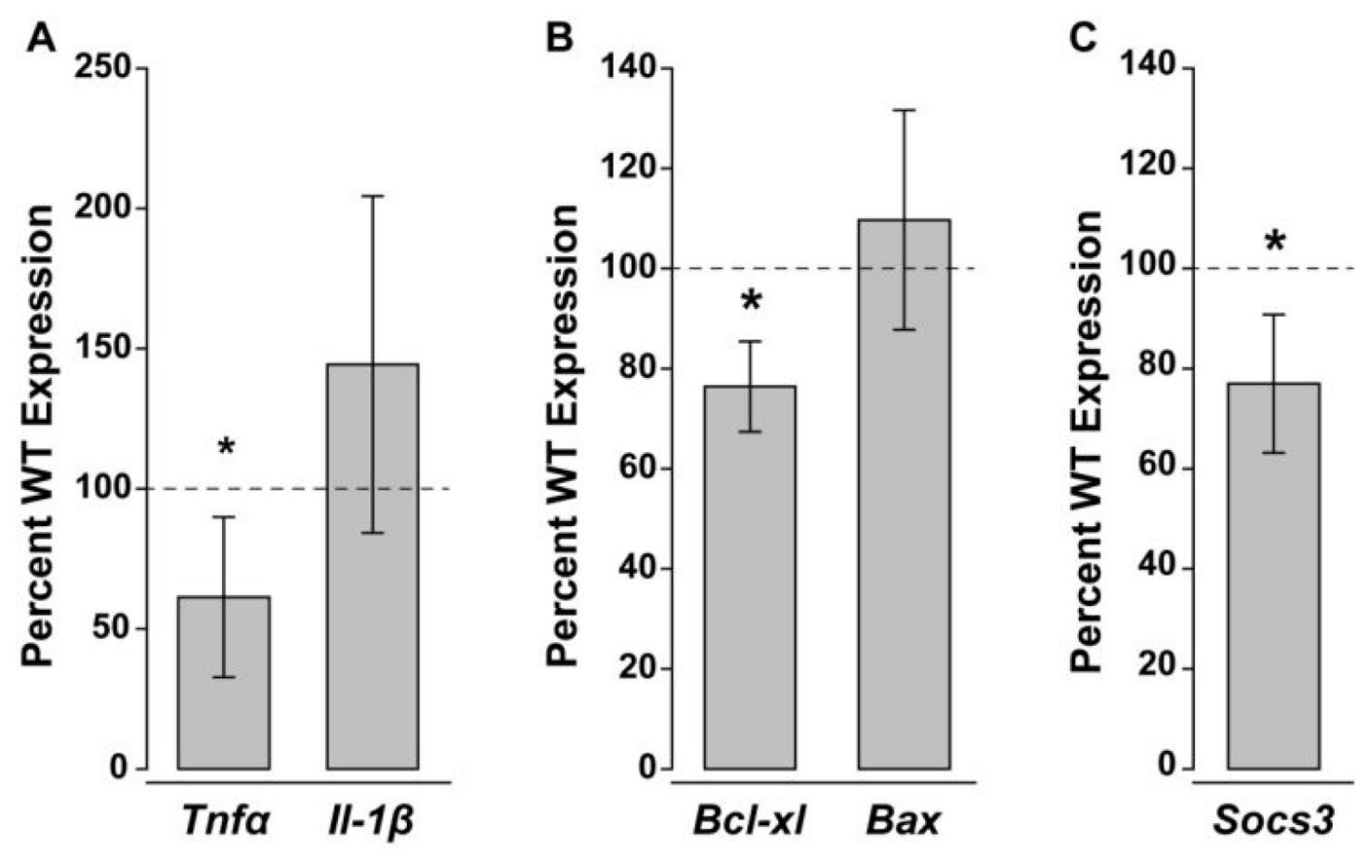
IL-6 deficiency alters baseline gene expression for neuroinflammation, cell health and gp130 regulatory signaling. Percent gene expression of (**A**) *Tnfα*, *Il-β* (**B**) *Bcl-xl*, *Bax* and (**C**) *Socs3* mRNA in the *IL-6−/−* retina normalized to WT expression levels (dotted line) via the ΔΔCt method. Asterisks indicate p<0.05.

To determine whether constitutive IL-6 deficiency and subsequent decreased expression of gp130 impact baseline parameters for neuroinflammation and cell health in retina, we examined mRNA expression of general neuroinflammatory factors as well as cell survival and regulatory factors associated specifically with gp130. For neuroinflammation outcomes we focused on two inflammatory factors associated with the initiation of inflammatory responses, tumor necrosis factor alpha (TNFα) and interleukin-1 beta (IL-1β). For cell health outcomes, we focused on two cell survival factors associated with promoting survival and initiating apoptosis, B-cell lymphoma-extra-large (Bcl-xl) and b-cell lymphoma-2 Associated X Protein (Bax). For regulatory signaling, we focused on suppressor of cytokine signaling -3(Socs3), which serves as a negative feedback mechanism for gp130 signaling. Using quantitative RT-PCR, we found that IL-6 deficiency reduced *Tnfα* mRNA expression by ~40%, as compared to WT expression levels (p<0.05; Figure 3A). In contrast, *Il-1β* mRNA expression was similar in retina from *IL-6−/−* and WT mice (p>0.05; Figure 3A). Like cytokine expression, IL-6 deficiency differentially altered gene expression associated with cell survival. In *IL-6−/−* retina, expression of *Bax* mRNA was 25% lower than in WT retina (p<0.05; Figure 3B). In contrast, we noted no significant difference in expression of *Bcl-xl* mRNA between *IL-6−/−* and WT retina (p>0.05; Figure 3B). Similarly, *Socs3* expression was also reduced by ~25% in *IL-6−/−* mice compared to WT mice (p<0.05; Figure 3C). These data indicate that constitutive IL-6 deficiency not only decreases gp130 expression levels, but also shifts baseline expression of neuroinflammatory, cell health and gp130 regulatory factors.

### IL-6 deficiency alters neuroinflammatory, cell health and gp130 regulatory responses to glaucoma stressors

IL-6-mediated changes in the constitutive expression of gp130 as well as the aforementioned downstream targets have the potential to significantly impact both the nature and magnitude of stress responses. Our previous work identified IL-6 signaling through gp130 as a relevant inflammatory pathway in glaucoma, a neurodegenerative disease affecting RGCs [9,10]. To determine whether IL-6-mediated changes in gp130 signaling impact the inflammatory, cell survival and gp130-related regulatory response of the retina to glaucomatous stressors, we examined gene expression of *Tnfα*, *lL-1β*,*Bcl-xl*, *Bax* and *Socs3* in the Microbead Occlusion Model of murine glaucoma [17,20]. As previously described, IOP was elevated in one eye for 4 weeks with a single intracameral injection of 15μm polystyrene beads The fellow eye received an equivalent volume of saline and served as the internal control [17].

**Figure 4:**
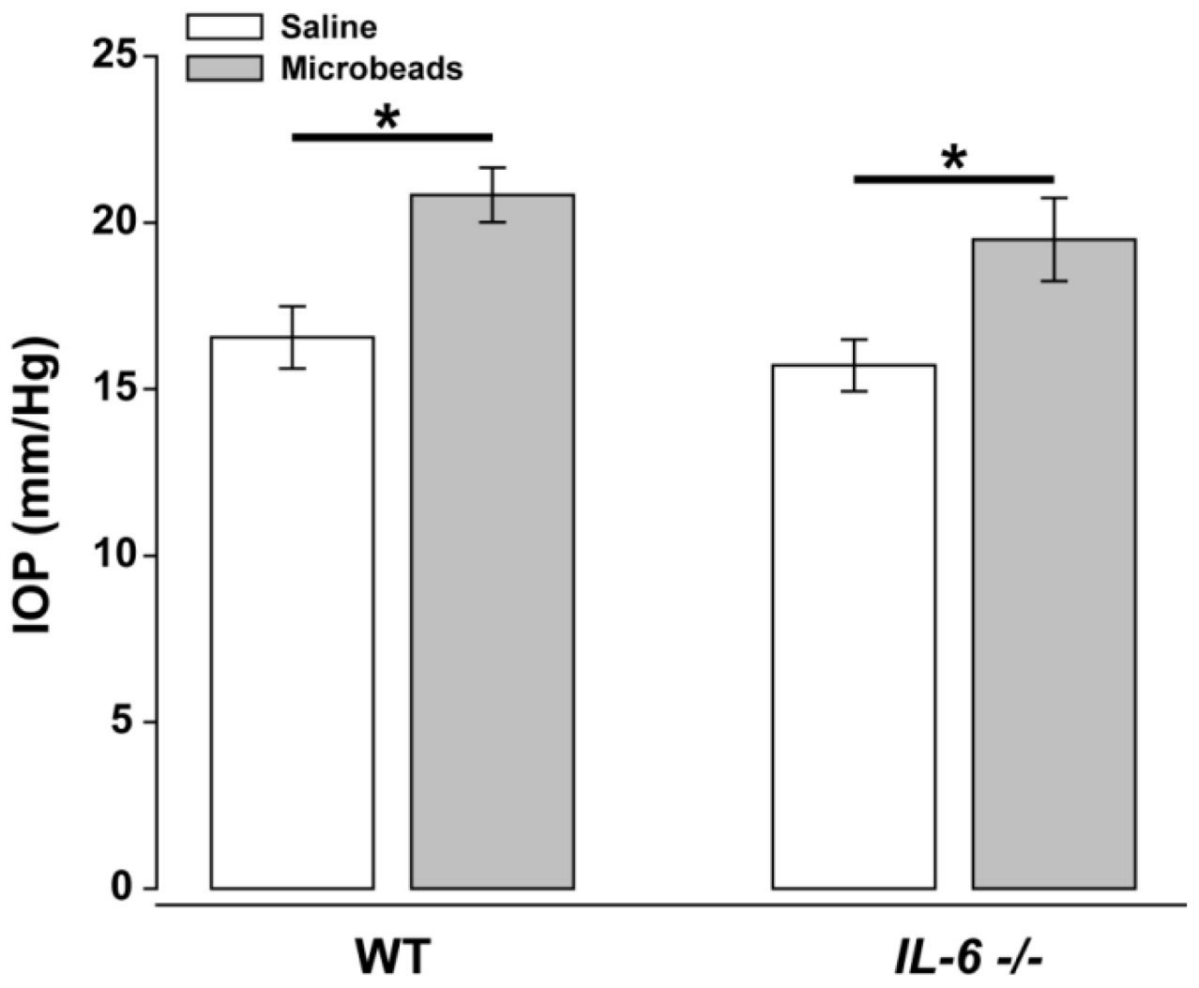
IL-6 deficiency does not impact microbead-induced IOP elevation. Average IOP of eyes injected with either saline (white) or microbeads (gray) over 4 weeks. Error bars represent standard error of the mean and asterisks indicate p<0.05.

**Figure 5:**
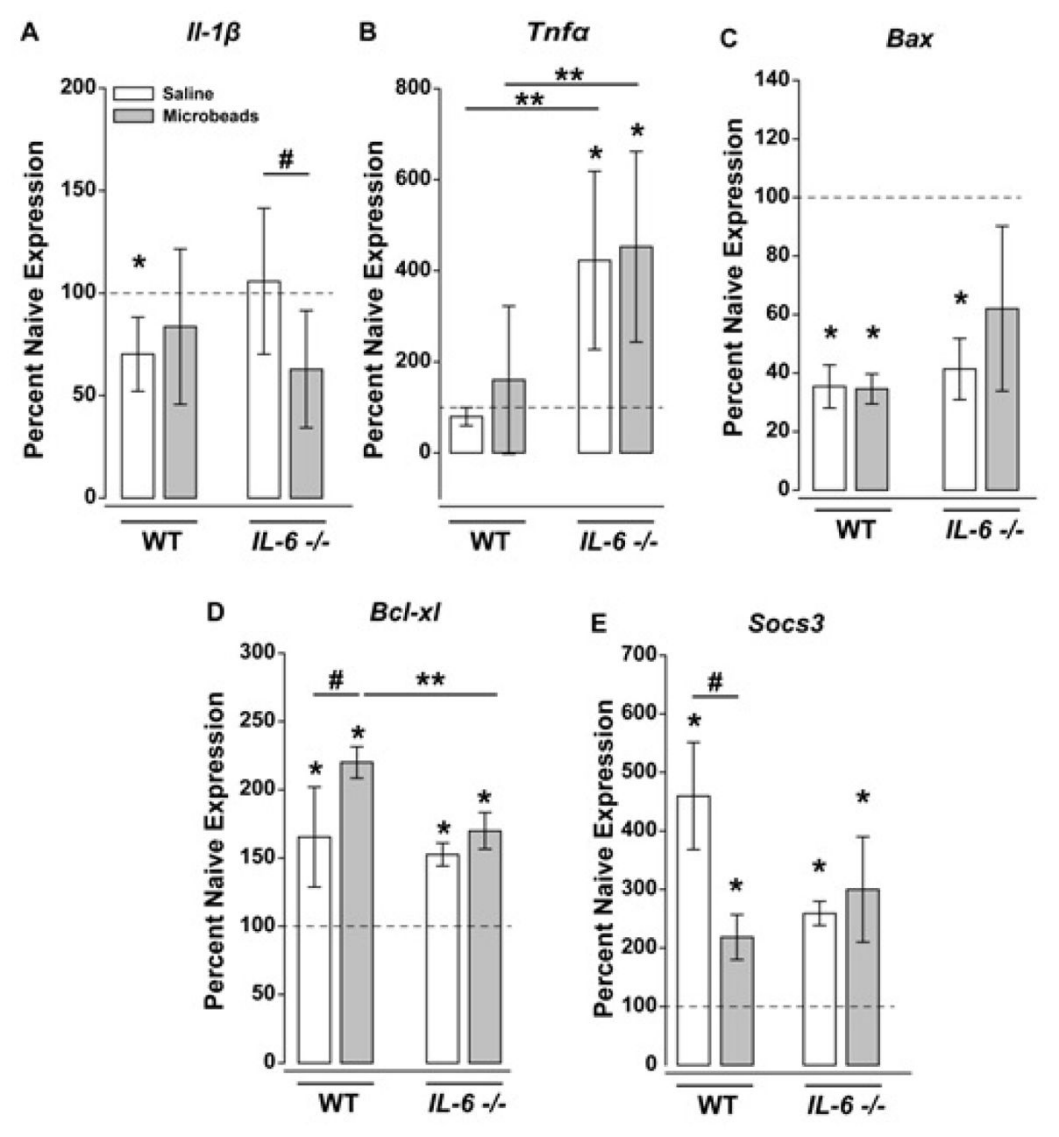
IL-6 deficiency influences glaucoma-related changes in gene expression of neuroinflammory, cell health and gp130 modulators. (**A–E**) Percent gene expression of (**A**) *Tnfα*, *Il-β* (**B**) *Bcl-xl* (**C**), *Bax* and (**D**) *Socs3* (**E**) mRNA in either WT or *IL-6−/−* eyes injected with saline (white) or microbeads (gray). mRNA levels were normalized to respective naive expression levels (dotted line) via the ΔΔCt method. Statistical significance (p<0.05) is indicated as follows: ^*^ naïve versus experimental, ^**^ between genotype comparison, ^#^ within genotype comparison.

Microbead injection increased IOP by ~25% in both WT and *IL-6−/−* mice, as compared to the contralateral saline-injected eye (p<0.05; Figure 4). For *Tnfα*, neither saline nor microbead injection altered *Tnfα* mRNA compared to naïve controls in WT mice (Figure 5B). In contrast, both saline- and microbead-injected eyes from *IL-6−/−* mice exhibit a 4-fold increase in *Tnfα* mRNA, compared to naïve *IL-6−/−* retinas and WT retinas exposed to either saline or microbead injection (p<0.05; Figure 5B). For *Il-1β*, elevated IOP did not alter mRNA levels in WT mice, as compared to saline-injected controls (p>0.05; Figure 5A). Similarly, neither saline-injection nor microbead-injection altered *Il-1β* mRNA in WT from naïve levels (p>0.05; Figure 5A). In contrast, elevated IOP reduced *Il-1β* mRNA by almost 40% in *IL-6−/−* mice, as compared to saline-injected *IL-6−/−* mice (p<0.05; Figure 5A). *Il-1β* mRNA expression in saline-injected *IL-6−/−* mice did not differ from that in naïve *IL-6−/−* mice or naïve, saline-injected and microbead-injected WT mice (p>0.05 for all; Figure 5A).

For pro-apoptotic gene *Bax*, both saline and microbead injections decreased mRNA levels by ~65% in WT mice, when compared to un-injected controls (p<0.05; Figure 5C). However, *Bax* mRNA expression did not differ between saline- and microbead-injected eyes in WT mice (p>0.05; Figure 5C). Similar decreases in *Bax* mRNA expression were noted for saline- (~40%) and microbead-injected (~60%; p < 0.05) *IL-6−/−* mice (Figure 5C). However, reductions in *Bax* mRNA associated with microbead-injection in *IL-6−/−* did not quite reach statistical significance (p=0.07; Figure 5C). For all conditions, *Bax* mRNA expression did not differ between WT and *IL-6−/−* mice (p>0.05 for all, Figure 5C). In contrast to *Bax*, expression of the anti-apoptotic gene *Bcl-xl* increased significantly with both saline and microbead injections in WT mice. In saline-injected eyes from WT mice, *Bcl-xl* mRNA increased approximately 1.5 fold, as compared to un-injected controls (p<0.05; Figure 5D). Elevated IOP increased *Bcl-xl* expression even further than saline-injection to levels 2.5-fold higher than un-injected controls (p<0.05; Figure 5D). Similarly, both saline and microbead injection increased *Bcl-xl* expression in *IL-6−/−* mice (p<0.05; Figure 5D). However, this increase was significantly lower in *IL-6−/−* mice than in WT mice (p<0.05; Figure 5D). No significant difference in *Bcl-xl* expression was noted between saline and microbead injected *IL-6−/−* mice (p>0.05; Figure 5D).

For *Socs3*, all conditions and genotypes exhibited an increase in mRNA expression, as compared to un-injected controls (p>0.05 for all; Figure 5E). The largest change was noted in saline-injected eyes from WT mice, which exhibited a 4.5-fold increase in *Socs3* expression compared to naïve WT mice (p>0.05; Figure 5E). Saline-injected *IL-6−/−* eyes and microbead-injected eyes from both WT and *IL-6−/−* exhibited *Socs3* expression that was ~ 2-fold higher than un-injected controls, but ~2.5-fold lower than saline-injected eyes from WT mice (p<0.05 for all; Figure 5E). Together, these data suggest that IL-6 signaling impacts specific components of stress-induced neuroinflammatory, cell health and gp130 regulatory signaling that alters not only the nature, but also the magnitude, of these stress responses.

## Discussion

The shared mechanism of signal transduction produces functional redundancy among IL-6 family members [1]. This functional redundancy impacts neuroinflammatory and cell health outcomes as well as regulatory signaling that mediates gp130 activity. Here we determined whether IL-6 may be relevant for establishing baseline parameters for neuroinflammatory, cell health and gp130-related regulatory signaling as well as the subsequent capacity for stress responses in these areas.

Using *IL-6−/−* mice, we determined whether constitutive IL-6 signaling plays a role in setting the baseline parameters for neuroinflammatory, cell health and gp130 regulatory signaling. Using q-RTPCR, we found that IL-6 deficiency decreased protein, but not mRNA levels, of its signal transducer gp130. This disparity between gp130 mRNA and protein levels could be due to deficits in translation or stability, which is documented in the regulation of gp130 protein levels [21–23]. Thus, IL-6 appears to mediate constitutive expression of gp130, which has the potential to impact signaling by other members of the IL-6 cytokine family. We also observed decreased expression of *Socs3* in *IL-6−/−* mice, which is consistent with studies in other tissues [24] and suggests that while gp130 availability is reduced, restraint on downstream signaling via JAK/STAT is lessened. These changes in gp130 regulation are accompanied by a reduction in mRNA levels for both *TNFα* and *Bax*, but not *IL-1β* or *Bcl-xl*. It is worth noting that previous work in heart tissue from *IL-6−/−* mice demonstrated similar decreases in *Bax* expression [25]. In contrast, previous work in brain did not detect any obvious differences in *Tnfα* or *Il-1β* expression in *IL-6−/−* mice [26,27]. It is likely that IL-6-dependent changes in TNFα noted here are tissue-specific, which would not be surprising given the high level of constitutive IL-6 expression in retina. Given that TNFα is associated with the initiation of inflammatory responses and Bax is a pro-apoptotic factor, our data suggests that IL-6 deficiency actually promotes an anti-inflammatory, pro-survival environment in which there is the potential for diminished gp130 signaling. However, this decrease in gp130 availability may be offset by lessened inhibition of STAT-mediated signaling.

A modified baseline of neuroinflammation, cell health and gp130 signaling has the potential to influence both the nature and magnitude of the stress response. In this context, we examined the same neuroinflammatory, cell health and gp130 regulatory factors in *IL-6−/−* and WT mice 4 weeks after induction of microbead-induced glaucoma. Regardless of genotype, we found that model induction (intracameral injection) resulted in the induction of several neuroinflammatory, cell health and gp130 regulatory factors. In most cases, the nature and the magnitude of the effects were similar between saline-injected and microbead-injected eyes. It is important to note that recent work in another inducible model of glaucoma reported the phenomenon of contralateral inflammation, in which the naïve fellow eye exhibited signs of neuroinflammation [28,29]. Since we performed saline and microbead injections (one in each eye) in the same animal, our results may be confounded by this phenomenon as well. To account for this issue, we analyzed our results with respect to un-injected, naïve controls and thus, treated saline injection as a separate experimental condition.

Our data indicate that while *TNFα* expression remained unaltered in WT mice, *IL-6−/−* mice exhibited a dramatic increase in *TNFα* expression following both saline and microbead injection. *IL-6−/−* specific increases in TNFα are reported in other models of stress/injury [30,31] and suggests that IL-6 may serve to diminish the TNFα response in glaucomatous retina, which is consistent with studies detailing the inhibitory effects of IL-6 signaling on TNFα [14]. Although IL-6 can also inhibit IL-1β [14], we found that *IL-6−/−* mice exhibited a decrease in IL-1β expression that was specific to microbead-injection (elevated IOP) and absent in WT mice and saline-injected *IL-6−/−* mice. This data suggests that other signaling pathways are likely more important for regulation of IL-1β in glaucoma. With respect to cell health, both WT and *IL-6−/−* exhibited decreased expression of *Bax* and increased expression of *Bcl-xl*. However, the Bcl-xl response was diminished in *IL-6−/−* mice versus WT mice, suggesting that the pro-survival response was blunted by IL-6 deficiency. Finally, *Socs3* expression was dramatically elevated in all experimental conditions for both WT and *IL-6−/−* mice. This suggests that a robust suppression of STAT signaling is part of the stress response to microbead-induced glaucoma. Interestingly, the most robust response was noted in saline-injected eyes from WT animals. Given that saline injection does not elevate IOP or induce RGC degeneration, this suggests that SOCS3 elevation may be particularly relevant for pre-emptive responses to sub-pathological insults.

Of particular importance for the interpretation of our findings in the Microbead Occlusion Model is the 4 week time point of analysis. In this model, RGC loss at 4 weeks is rather minimal (less than 25%) and most of the pathological changes are consistent with early stages of degeneration [17,20,32]. As such, our results indicate that, among the factors tested, the overall nature of the inflammatory and cell health response is geared towards the promotion of cell survival and inhibition of inflammatory signaling. With respect to IL-6, our results indicate that, while constitutive IL-6 deficiency appears to promote an anti-inflammatory environment and pro-survival, this deficiency permits an exaggerated TNFα response with exposure to stressors and diminishes expression of anti-apoptotic factors. Overall, our findings support a role for IL-6 in setting baseline parameters for neuroinflammatory, cell health and gp130 regulatory signaling that can impact the nature and magnitude of retinal responses to glaucoma-related stressors.
